# Supplementary material for: Quantifying hot topic dynamics in scientific literature: An information-theoretical approach
Source: PLoS One. 2025 Jul 8;20(7):e0327793. doi: 10.1371/journal.pone.0327793 (PMC12237269; doi:10.1371/journal.pone.0327793)
Supplement: S1 Appendix — (PDF) [file pone.0327793.s001.pdf]

Correction to distances from changing data set size. We want to answer the question: Do we have to take into account the growing size of the dataset $N(t)$ when comparing the distances $d(X < Y, t)$ for the same pair of target concepts A and B but for different time frames (that is, when $N(t_2) - N(t_1) = \Delta N > 0$ for $t_2 > t_1$ and $t_0 = \text{const}$)?

To answer this question, we have to consider two cases: first, when ΔN does not have any documents where the target concepts are mentioned (that is, $\Delta N_A = 0$ and $\Delta N_B = 0$); and second, when $\Delta N_A > 0$ and $\Delta N_B > 0$ so concepts appear in the new documents.

In Fig S1 Fig, we calculate the dependence of $d(N)$ when N continuously increases from an initial value $N_0 = 10000$, while the newly added documents do not mention the target concepts. In this scenario, the probability $P(0,0)$, which represents the fraction of documents in which neither of the two concepts appears, increases its relative weight in correspondence to other probabilities as the dataset grows. Since these new documents contribute equally to both the numerator and the denominator of $P(0,0)$, this probability approaches asymptotically a higher value as N increases.

Conversely, all other probabilities $P(k,m)$ for $k, m > 0$, representing the co-occurrence of concepts in meaningful documents, are affected differently. The extra documents contribute only to the denominator of these terms, effectively "reducing" their values. As a result, the mutual information and entropy measures derived from these probabilities become increasingly "dominated by the growth of $P(0,0)$", which skews the calculated distance metrics over time.

We find that the distance $d(N)$ as a function of the size of the data set behaves following the model:

$$d(a, \alpha, l; N) = \frac{a}{\log N}\left(1 + \alpha(\log N + L)\right) \simeq b + \frac{c}{\log N} \quad \text{if} \quad \Delta N \to 0. \tag{5}$$

As we can see in S1 Fig, the model dependence obtained for a fixed value of the parameters $a > 0, \alpha > 0, L < 0$ represents a slow and predictable behavior when d gradually decreases over time. The rate of change in d is higher if a pair of concepts initially was closer to each other. At small ΔN, the function $d(N)$ can be approximated by the linear function (see Eq.(5)) with parameters $b = a\alpha > 0$ and $c = a + bL < 0$. The parameter b shows a high sensitivity to changes in the number of 'relevant' documents with non-zero frequencies (i.e. for which $k, m > 0$) for the target concepts. It increases if the number of documents that mention both concepts is decreasing, which, as expected, drives the distance $d \to 1$ (see S1 Figb). And b is decreasing as the number of documents mentioning at least one of the concepts is decreasing (see S1 Figc and d) while the number of documents where concepts co-occur is kept constant. The parameter c denotes the slope of $d(N)$ as N increases, showing a weak dependence on the variation in the number of relevant documents, and it is mainly a function of N.
