# Supplementary figures and images for: Quantifying hot topic dynamics in scientific literature: An information-theoretical approach
Source: PLoS One. 2025 Jul 8;20(7):e0327793. doi: 10.1371/journal.pone.0327793 (PMC12237269; doi:10.1371/journal.pone.0327793)

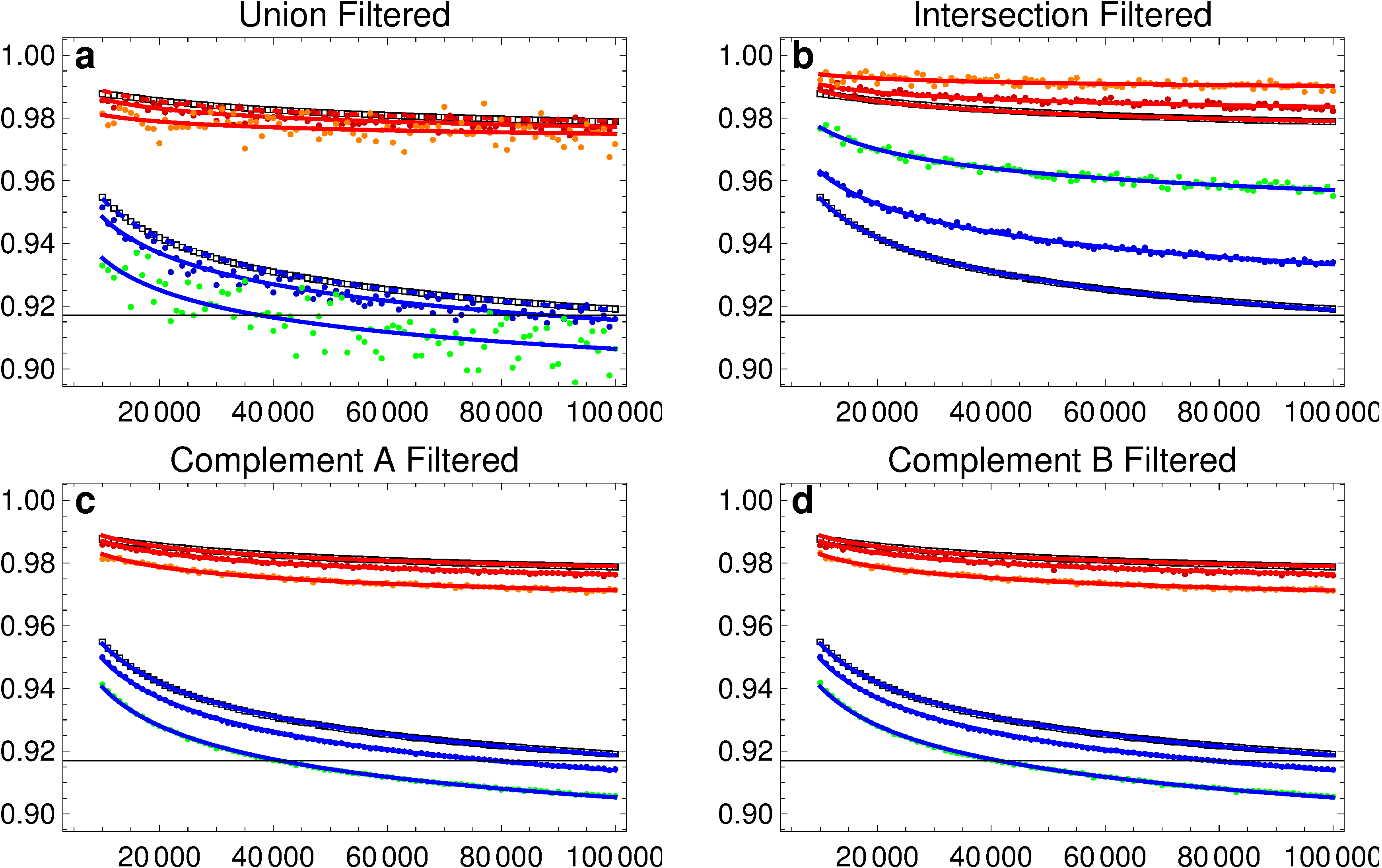

Supplement: S1 Fig — For two target concept pairs –“Pair 1” (blue palette) and “Pair 2” (red palette) – the distance d(N) is calculated over a range of data set sizes from N=10000 to N=100000, with N increasing in steps of 1000. In these experiments, only the number of documents with zero citation frequencies for the target pair is increased. For each pair, three series of d(N) data points are calculated and fitted with the model in Eq (5), corresponding to the following scenarios: Baseline: All relevant documents (that is, those with k,m>0) are retained, with the baseline data shown as empty squares and the fitted model plotted as a dashed curve.20% Removal: At each step, 20% of the relevant documents are randomly removed from the union of all papers that mention at least one of the target concepts, while the background (documents with zero citations for the target pair) increases by 1000.50% Removal: Similarly, 50% of the relevant documents are randomly removed at each step (green points for Pair 1 and orange points for Pair 2). Each subfigure represents a different target for random document removal: subfigure (a) shows removal from the union of all papers mentioning either concept; subfigure (b) shows removal from the intersection (i.e. only those documents that mention both concepts); and subfigures (c) and (d) show removal from the complement (i.e. only those documents that mention a single concept from the target pair). To demonstrate that the dynamics of the conceptual distance d for a given pair of concepts A and B is weakly influenced by the size of the data set N and the increasing number of background documents with zero concept frequencies, and that the primary driver of the decrease in conceptual distance is the number of documents mentioning both concepts NAB, we present in S2 Fig the time series for key metrics observed for multiple pairs of concepts in the studied dataset. Key metrics include NVI distance d, mutual information, joint entropy, total size of the dataset N [file pone.0327793.s002.tif]

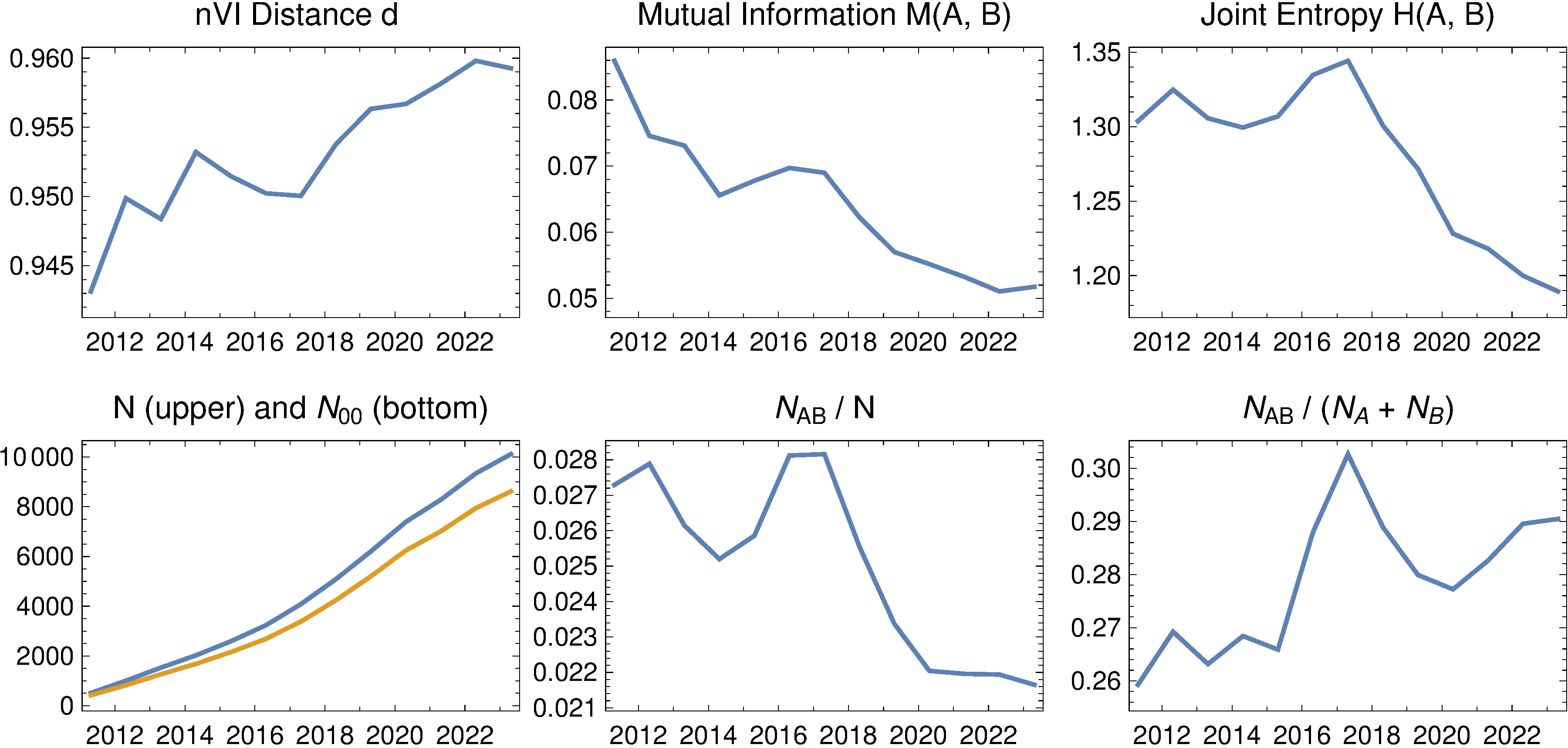

Supplement: S2 Fig — Time series of key metrics that illustrate the relationship between conceptual distance d, the size of the data set N, relevant documents and the number of background documents N00. The plots present the dynamics of the NVI distance d between concepts A and B, their mutual information M(A,B), joint entropy H(A,B), total dataset size N, the number of background documents N00 (documents that do not mention these concepts), the ratio of documents mentioning both concepts NAB to the total dataset size, and the ratio of NAB to the sum of documents mentioning only one of the concepts, NA+NB. The results indicate that while dataset expansion alters global entropy-based measures, the conceptual distance is predominantly shaped by the number of co-occurring mentions of the concepts, underscoring the role of shared contextual usage rather than dataset size alone. The analysis of conceptual distance dynamics highlights that the expansion of the dataset, as reflected in the dynamics of N or N00 in S2 Fig, does not affect the behavior of the distance d in the manner predicted by the model in Eq (5) when only the number of background documents of type N 00 increases. This finding suggests that the conceptual distance is primarily dependent on the number of documents in which the target concepts co-occur, which is evident from the behavior of the ratios NAB/N and NAB/(NA+NB): as these ratios increase, the distance d decreases. Furthermore, the effect of random variations on the number of relevant documents, as observed in S1 Fig, reinforces this conclusion. The random removal of relevant documents affects the dynamics of d in a way that is consistent with its dependence on the presence of concept co-occurrence, rather than merely on dataset size and the number of background documents. These results collectively indicate that meaningful conceptual proximity is driven by shared document contexts rather than by the absolute number of documents in the dataset. (TIF) [file pone.0327793.s003.tif]

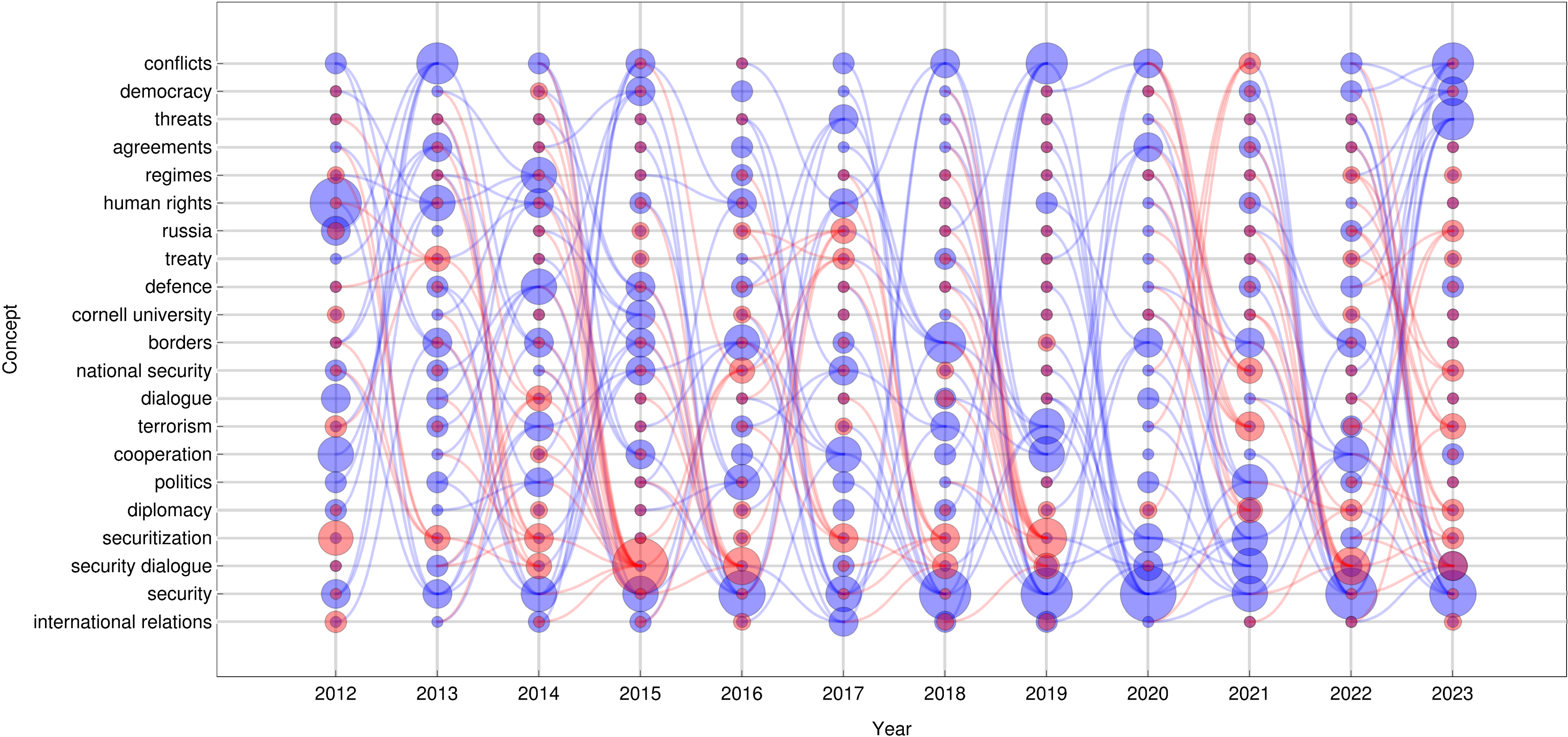

Supplement: S3 Fig — The bubble-flow graph illustrates the evolution of topics related to the ‘International Relations’ seed concept. The visualization highlights topic convergence (red curves and bubbles), where concepts move closer to a hub concept in terms of the d metric, and topic divergence (blue curves and bubbles), where concepts become less related to the hub concept over time. The size of the bubbles reflects the degree centrality of each node in the MST-based topic network. (TIF) [file pone.0327793.s004.tif]

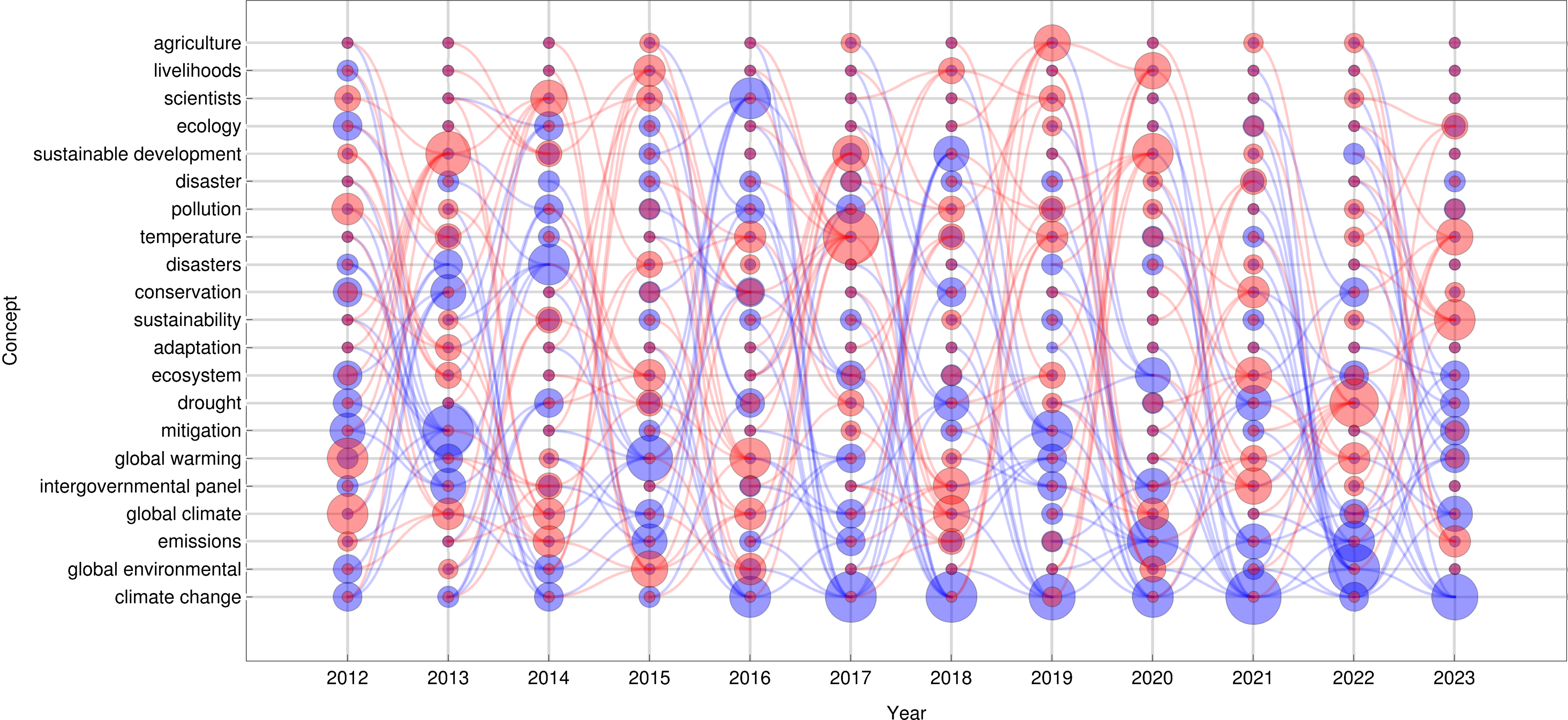

Supplement: S4 Fig — The bubble-flow graph illustrates the evolution of topics related to the ‘Climate change’ seed concept. The visualization highlights topic convergence (red curves and bubbles), where concepts move closer to a hub concept in terms of the d metric, and topic divergence (blue curves and bubbles), where concepts become less related to the hub concept over time. The size of the bubbles reflects the degree centrality of each node in the MST-based topic network. (TIF) [file pone.0327793.s005.tif]

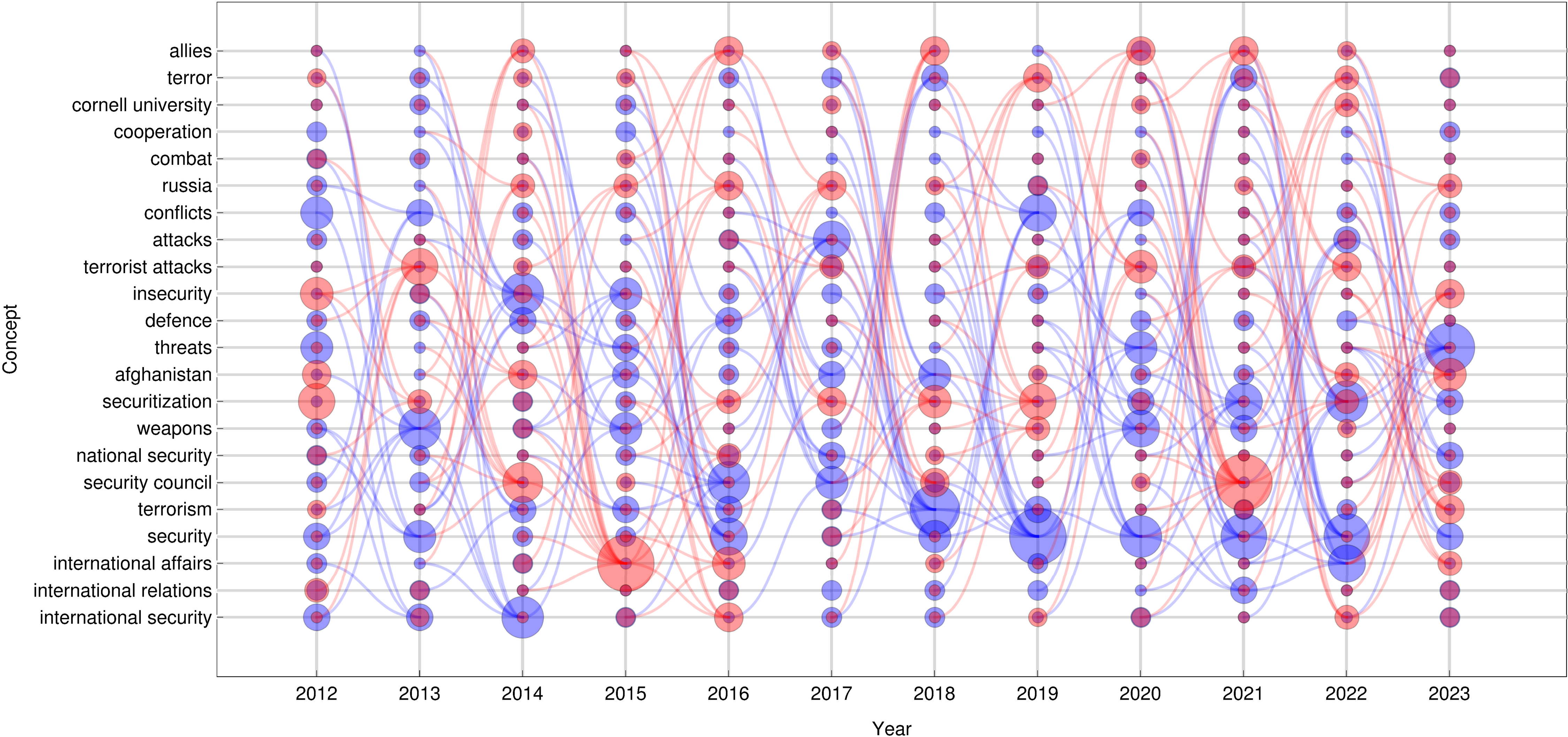

Supplement: S5 Fig — The bubble-flow graph illustrates the evolution of topics related to the ‘International Security’ seed concept. The visualization highlights topic convergence (red curves and bubbles), where concepts move closer to a hub concept in terms of the d metric, and topic divergence (blue curves and bubbles), where concepts become less related to the hub concept over time. The size of the bubbles reflects the degree centrality of each node in the MST-based topic network. (TIF) [file pone.0327793.s006.tif]

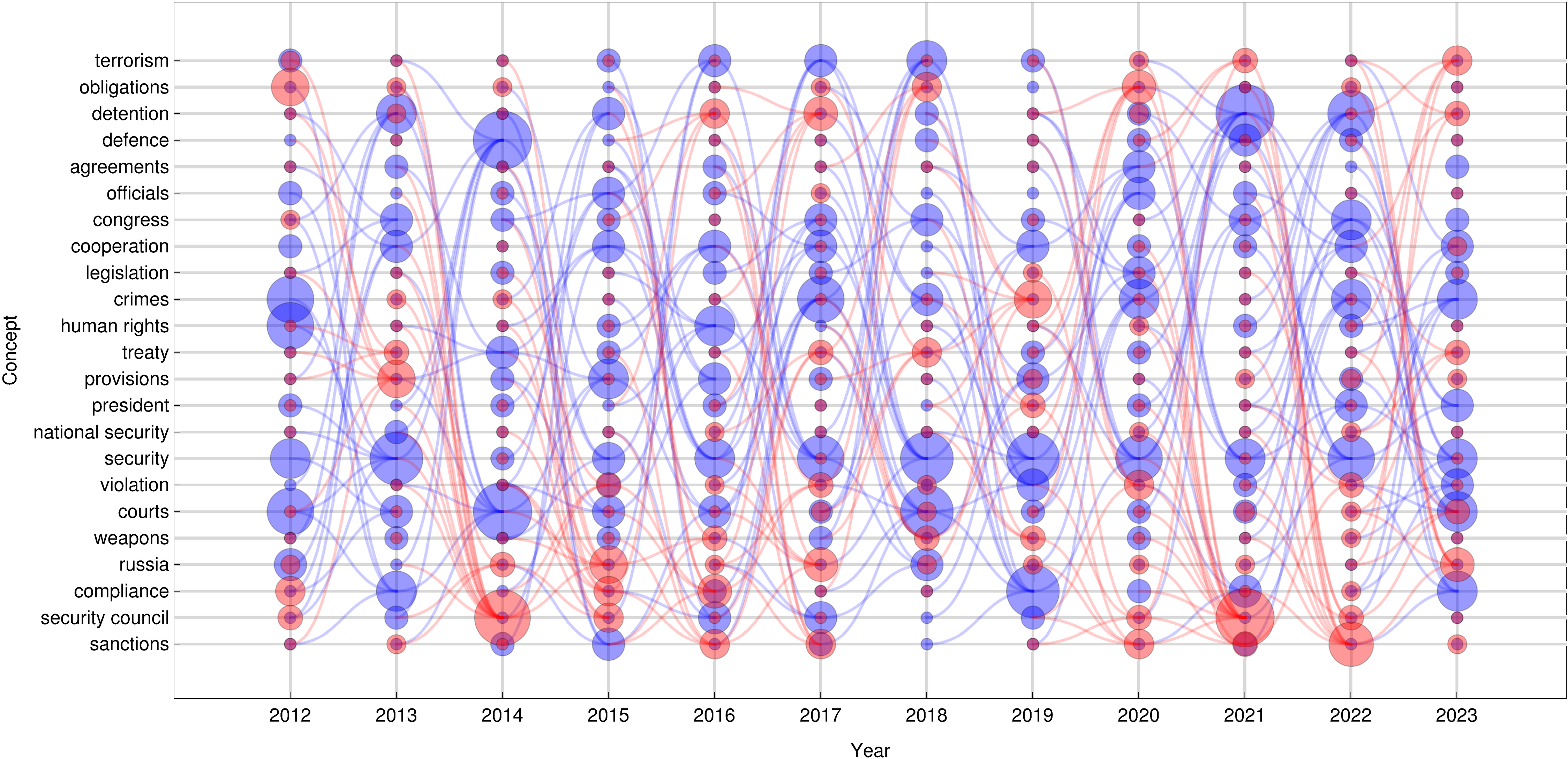

Supplement: S6 Fig — The bubble-flow graph illustrates the evolution of topics related to the ‘Sunctions’ seed concept. The visualization highlights topic convergence (red curves and bubbles), where concepts move closer to a hub concept in terms of the d metric, and topic divergence (blue curves and bubbles), where concepts become less related to the hub concept over time. The size of the bubbles reflects the degree centrality of each node in the MST-based topic network. (TIF) [file pone.0327793.s007.tif]

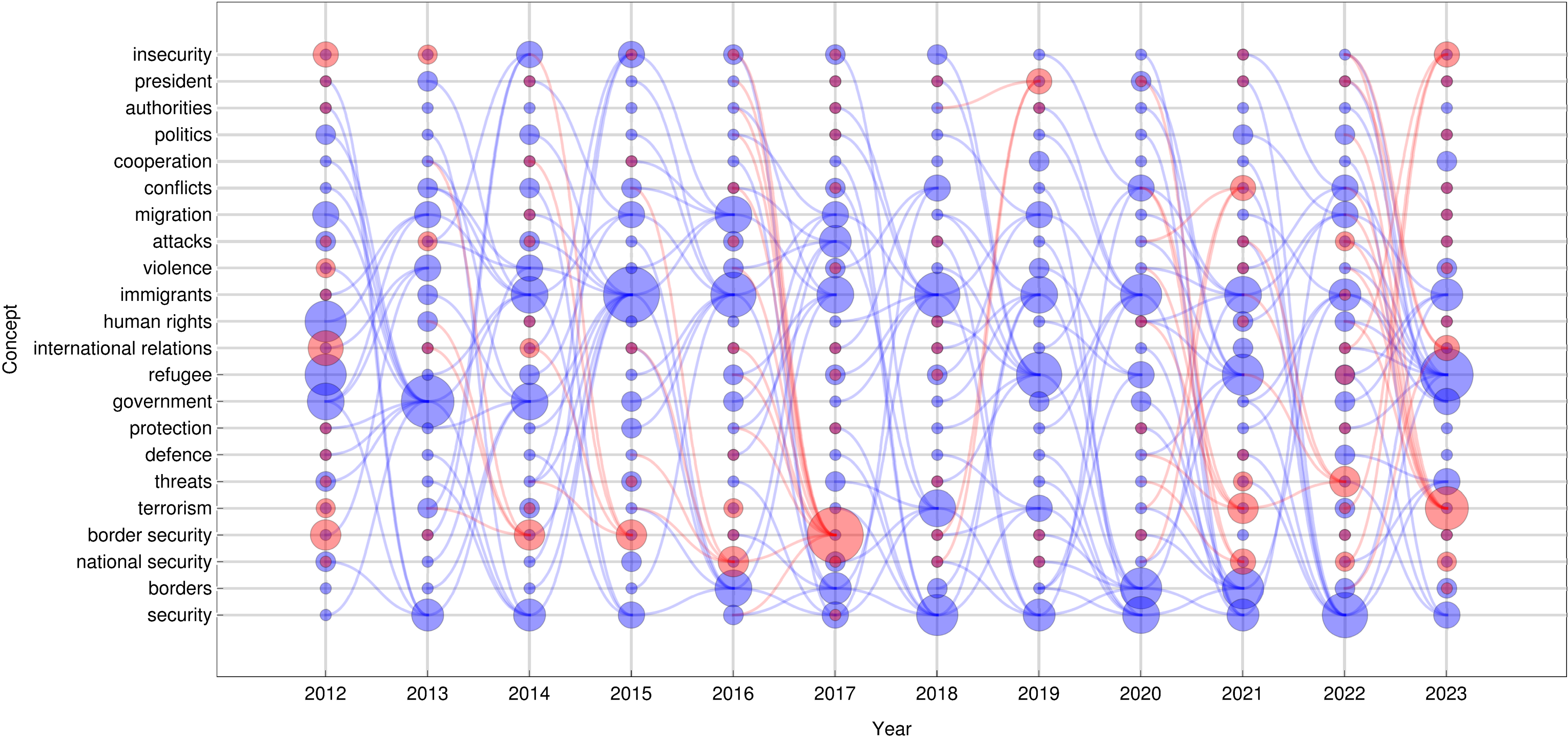

Supplement: S7 Fig — The bubble-flow graph illustrates the evolution of topics related to the ‘Security’ seed concept. The visualization highlights topic convergence (red curves and bubbles), where concepts move closer to a hub concept in terms of the d metric, and topic divergence (blue curves and bubbles), where concepts become less related to the hub concept over time. The size of the bubbles reflects the degree centrality of each node in the MST-based topic network. (TIF) [file pone.0327793.s008.tif]
