# Supplementary material for: Quantifying hot topic dynamics in scientific literature: An information-theoretical approach
Source: PLoS One. 2025 Jul 8;20(7):e0327793. doi: 10.1371/journal.pone.0327793 (PMC12237269; doi:10.1371/journal.pone.0327793)
Supplement: Attachment — Submitted filename: Response to Reviewers.docx [file pone.0327793.s009.docx]

# Response to Reviewers and Editor

We sincerely thank the reviewers and the editor for their constructive comments and valuable suggestions, which helped improve the quality and clarity of the manuscript.

## Editor Comments

1. Please ensure that your manuscript meets PLOS ONE’s style requirements, including those for file naming.

Response:
All formatting and file naming requirements have been verified and are now fully satisfied.

2. The grant information provided in the ‘Funding Information’ and ‘Financial Disclosure’ sections do not match. Please ensure consistency.

Response:
The following unified statement is now used in both sections:
“The author received no specific funding for this work.”

3. We are unable to open your Supporting Information and figure files (S1 Fig to S7 Fig and Fig 1, Fig 2). Please revise and re-upload.

Response:
All supplementary files are provided in `.eps` format. I have verified that the files are not corrupted and can be opened using online EPS viewers (e.g., https://jumpshare.com/viewer/eps). Figures Fig 1 and Fig 2 are also available at the end of the submission file in EPS format for visual inspection.

## Reviewer #1 Comments

1. “Please explain the researcher’s conclusions and what he addressed within the research gap of the work presented in the abstract.”

Response:
The abstract has been revised to clearly state the research gap, the proposed solution, and the main findings.

2. “Ensure that references are included for all equations in the research.”

Response:
References have been added before the introduction of formulas (1), (2), and (3). Formulas (4) and (5) are original contributions by the author and are noted as such.

3. “The research paper lacks tables and charts that reflect the researcher’s reading and summary of the reviewed research, and does not highlight the strengths and weaknesses of previous research.”

Response:
We appreciate this valuable comment. While comparative tables are useful in methodological surveys, our manuscript is not intended as a review or benchmark of topic detection methods. Rather, our contribution is a complementary approach that focuses on within-topic structural dynamics and conceptual proximity analysis, which are not the primary targets of conventional topic modeling techniques (e.g., LDA, DTM, NTMs).

Our method can be applied in conjunction with topic models. For example, once a topic is identified via LDA, our framework can track semantic drift, detect convergence hubs, and evaluate the influence of knowledge producers on concept evolution. To clarify this distinction, we have added a paragraph to the Introduction section.

4. “Add references published in 2025.”

Response:
We have added a recent reference: Tan Z, D’Souza J. Bridging the Evaluation Gap: Leveraging Large Language Models for Topic Model Evaluation. arXiv preprint. 2025; arXiv:2502.07352. It is cited in the Introduction section.

## Reviewer #2 Comments

1. “The current state of research on topic modeling is discussed, but important studies such as Structural Topic Modeling (STM) by Roberts et al. (2014) are missing.”

Response:
We have added discussion of STM and relevant citations to the Introduction section.

2. “The temporal development of topics should be linked back more clearly to specialized discourses.”

Response:
We have expanded the Results section with contextual interpretations for the observed evolution of “cybersecurity” and “artificial intelligence” as key concepts.

3. “No working link was found to the GitHub repository; please ensure the code is available and documented.”

Response:
The code and data are accessible via the Zenodo repository listed in the Data Availability section: https://doi.org/10.5281/zenodo.14948128, which includes a link to the GitHub repository: https://github.com/ArtemEsper/TopicGeometry. The repository is documented and includes the necessary scripts and usage instructions.
